# Cerebro-cerebellar motor networks in clinical subtypes of Parkinson’s disease

**DOI:** 10.1038/s41531-022-00377-w

**Published:** 2022-09-06

**Authors:** Silvia Basaia, Federica Agosta, Alessandro Francia, Camilla Cividini, Roberta Balestrino, Tanja Stojkovic, Iva Stankovic, Vladana Markovic, Elisabetta Sarasso, Andrea Gardoni, Rosita De Micco, Luigi Albano, Elka Stefanova, Vladimir S. Kostic, Massimo Filippi

**Affiliations:** 1 Neuroimaging Research Unit, Division of Neuroscience, IRCCS San Raffaele Scientific Institute, Milan, Italy; 2 Neurology Unit, IRCCS San Raffaele Scientific Institute, Milan, Italy; 3 Vita-Salute San Raffaele University, Milan, Italy; 4 Clinic of Neurology, Faculty of Medicine, University of Belgrade, Belgrade, Serbia; 5 Laboratory of Movement Analysis, San Raffaele Scientific Institute, Milan, Italy; 6 Department of Advanced Medical and Surgical Sciences, University of Campania “Luigi Vanvitelli”, Napoli, Italy; 7 Neurophysiology Service, IRCCS San Raffaele Scientific Institute, Milan, Italy; 8 Neurorehabilitation Unit, IRCCS San Raffaele Scientific Institute, Milan, Italy

**Keywords:** Parkinson's disease, Predictive markers

## Abstract

Parkinson’s disease (PD) patients can be classified in tremor-dominant (TD) and postural-instability-and-gait-disorder (PIGD) motor subtypes. PIGD represents a more aggressive form of the disease that TD patients have a potentiality of converting into. This study investigated functional alterations within the cerebro-cerebellar system in PD-TD and PD-PIGD patients using stepwise functional connectivity (SFC) analysis and identified neuroimaging features that predict TD to PIGD conversion. Thirty-two PD-TD, 26 PD-PIGD patients and 60 healthy controls performed clinical/cognitive evaluations and resting-state functional MRI (fMRI). Four-year clinical follow-up data were available for 28 PD-TD patients, who were classified in 10 converters (cTD-PD) and 18 non-converters (ncTD-PD) to PIGD. The cerebellar seed-region was identified using a fMRI motor task. SFC analysis, characterizing regions that connect brain areas to the cerebellar seed at different levels of link-step distances, evaluated similar and divergent alterations in PD-TD and PD-PIGD. The discriminatory power of clinical data and/or SFC in distinguishing cPD-TD from ncPD-TD patients was assessed using ROC curve analysis. Compared to PD-TD, PD-PIGD patients showed decreased SFC in temporal lobe and occipital lobes and increased SFC in cerebellar cortex and ponto-medullary junction. Considering the subtype-conversion analysis, cPD-TD patients were characterized by increased SFC in temporal and occipital lobes and in cerebellum and ponto-medullary junction relative to ncPD-TD group. Combining clinical and SFC data, ROC curves provided the highest classification power to identify conversion to PIGD. These findings provide novel insights into the pathophysiology underlying different PD motor phenotypes and a potential tool for early characterization of PD-TD patients at risk of conversion to PIGD.

## Introduction

Parkinson’s disease (PD) is a neurodegenerative disease characterized by a combination of motor and non-motor symptoms^1^. The clinical picture of PD can be highly heterogeneous, therefore different subtypes have been proposed. A classic definition based on motor symptoms and signs classified patients as tremor-dominant (PD-TD) or the postural-instability-and-gait-disorder (PD-PIGD) motor subtype^2^. Beyond motor features, PD-TD and PD-PIGD patients are characterized by distinctive clinical features and prognosis. The presence of gait and balance disturbances in PIGD influences loss of autonomy and risk of falls, one of the major determinants of morbidity and mortality in PD^3,4^. Moreover, PD-TD patients are characterized by slower progression, while PD-PIGD patients tend to exhibit a rapid decline of motor functions as well as cognition^5^. Importantly, PD-TD patients can convert into PD-PIGD subtype along the disease course, although the mechanisms underlying this evolution have not been elucidated yet^6^. Therefore, identifying early pathophysiological hallmarks and biomarkers of PD motor subtypes is a clinical and research priority, due to the possible association with different disease mechanisms, prognostic implications, and early pharmacological and non-pharmacological therapeutic indications^7^.

Functional MRI (fMRI) studies suggest that PD-TD patients have greater cerebello-thalamo-cortical (CTC) circuitry dysfunction, while PD-PIGD patients show deficits within the striato-thalamo-cortical network^8–14^. More recently, an increasing body of evidence suggests that the cerebellum plays a central function in PD pathophysiology, besides its broadly recognized role in the pathogenesis of tremor, hinting its involvement into other PD signs, including gait disturbances and non-motor symptoms^15^. Elucidating the distinct involvement of cerebellum in PD-TD and PD-PIGD may provide support for PD motor subtype differences.

Against this background, we characterized the involvement of CTC circuits in PD-TD and PD-PIGD patients using a novel network analysis, i.e., stepwise functional connectivity (SFC)^16^. In the last few years, graph analysis has been applied to PD thanks to its ability to assess at various disease stages how pathological changes in one network may alter structure and/or function of other brain regions^17,18^. Only one study so far has applied graph theory analysis to PD motor subtypes demonstrating widespread network alterations in both PD groups relative to controls, with PD-PIGD patients reporting more disrupted hubs in the cerebellum compared to PD-TD^19^. With such a framework, the first aim of this study was to detect, using SFC, which parts of the brain are connected with different cerebellar-seed regions not only through direct paths (i.e., one‐step functional distance), but also through indirect connections that involve a varying number of “link‐step” distances. Furthermore, we identified similar and divergent SFC alterations in PD-TD and PD-PIGD. Finally, we aimed to define fMRI features in the PD-TD group that predict the evolution to a PD-PIGD phenotype within 4 years of follow-up.

## Results

**Fig. 1:**
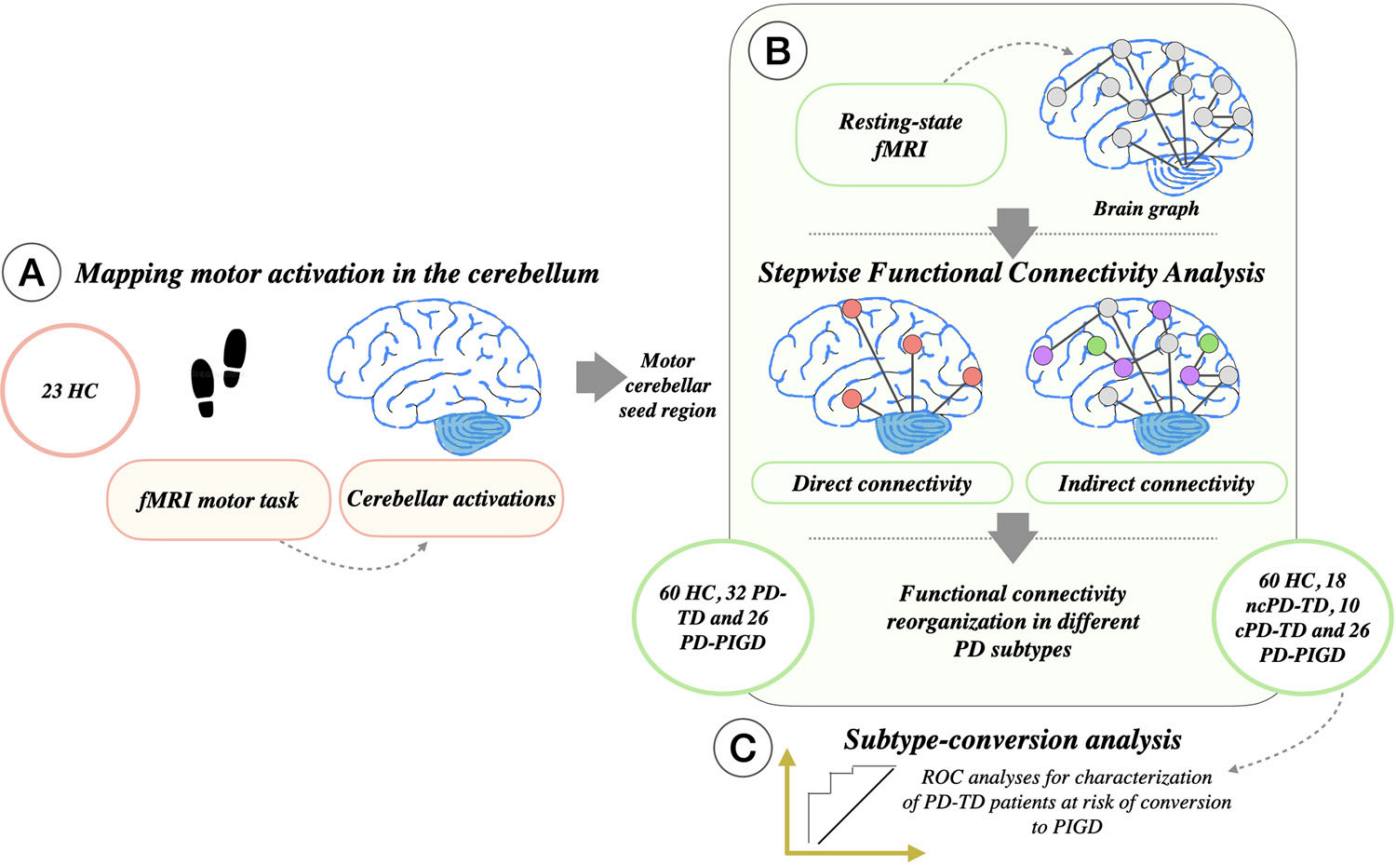
Study framework. **A** Mapping motor activation in the cerebellum. **B** Cortical connectivity diagram of a selected area in the cerebellum using stepwise connectivity analysis. Cortical maps show characterization of direct and indirect functional connectivity from motor cerebellar seed region. Functional connectivity reorganization was then evaluated in different PD subtypes. **C** ROC analyses for characterization of PD-TD patients at risk of conversion to PIGD. c, converter; fMRI, functional MRI; HC, healthy controls; nc, non-converter; PD-TD, Parkinson’s disease tremor dominant; PD-PIGD, Parkinson’s disease with postural instability and gait disorders.

Figure 1 reports briefly the study framework: (1A) Mapping motor activation in the cerebellum; (1B) Cortical connectivity diagram of a selected area in the cerebellum using SFC; (1C) Receiver operating characteristic (ROC) analyses for characterization of PD-TD patients at risk of conversion to PIGD.

### Demographic and clinical results

Demographics and clinical/cognitive characteristics are reported in Table 1 and Supplementary Tables 1 and 3. There was no difference between PD groups and controls in terms of age, sex, education, and handedness. The clinical characteristics of the two PD motor subtypes were quite homogenous except for Axial and Rigidity Unified Parkinson’s Disease Rating Scale (UPDRS)-III subscores that were higher in the PD-PIGD group. Concerning cognitive data, both PD patients performed worse compared to healthy controls in few cognitive domains. Furthermore, PD-TD patients had significant worse ACE-R language score relative to controls. No cognitive difference was found between the two PD groups.

**Table 1:** Demographic and clinical characteristics of healthy controls, PD-TD and PD-PIGD patients.

Variables	HC	PD-TD subtype	PD-PIGD subtype	p: HC *vs* PD-TD	p: HC *vs* PD-PIGD	p: PD-PIGD *vs* PD-TD
*N*	60	32	26			
*Demographic and general clinical variables*						
Age at MRI [years]	61.79 ± 8.9 (46.14–77.72)	60.96 ± 7.22 (43.43–75.87)	61.73 ± 5.28 (48.45–71.42)	1.00	1.00	1.00
Sex [men/women]	29 (48.3)/31 (51.7)	19 (59.4)/13 (40.6)	10 (38.5)/16 (61.5)	0.38	0.48	0.19
Education [years]	13.52 ± 2.57 (8.00–16.00)	12.88 ± 2.69 (7.00–20.00)	12.62 ± 2.65 (8.00–17.00)	0.84	0.47	1.00
Handedness [right/left/both]	46 (92)/4 (8)/0 (0)	31 (96.9)/1 (3.1)/0(0)	26 (92.0)/0 (0)/0 (0)	0.64	0.29	1.00
Age at onset [years]	–	59.13 ± 7.66 (42.50–73.00)	58.54 ± 6.23 (42.00–70.00)	–	–	0.75
PD duration [years]	–	1.83 ± 1.74 (0.14–7.57)	3.20 ± 4.74 (0.00–23.94)	–	–	0.14
Family history [No/Yes]	–	22 (68.8)/10 (31.3)	22 (84.6)/4 (15.4)	–	–	0.22
Side of onset [right/left/both]	–	20 (62.5)/12 (37.5)/0(0)	16 (61.5)/10 (38.5)/3 (3.3)	–	–	1.00
Levodopa equivalent daily dose [mg]	–	252.97 ± 266.97 (0.00–970.00)	342.60 ± 233.38 (0.00–1000.00)	–	–	0.18
*Clinical motor variables*						
Hoehn & Yahr	–	1.16 ± 0.47 (1.00–3.00)	1.12 ± 0.43 (1.00–3.00)	–	–	0.73
UPDRS Total	–	28.53 ± 15.43 (11.00–81.00)	31.00 ± 11.82 (17.00–76.00)	–	–	0.51
UPDRS II Total	–	5.72 ± 4.38 (0.00–19.00)	7.12 ± 4.01 (2.00–20.00)	–	–	0.22
UPDRS III Total	–	17.88 ± 10.18 (10.00–52.00)	17.96 ± 8.77 (10.00–52.00)	–	–	0.97
*UPDRS III Axial*	–	1.53 ± 1.50 (0.00–7.00)	2.58 ± 1.30 (1.00–7.00)	–	–	**0.01**
*UPDRS III Bradykinesia*	–	7.31 ± 5.27 (3.00–25.00)	8.92 ± 5.11 (5.00–27.00)	–	–	0.25
*UPDRS III Rigidity*	–	2.63 ± 1.62 (0.00–8.00)	3.89 ± 2.36 (2.00–13.00)	–	–	**0.02**
UPDRS IV Total	–	0.22 ± 0.94 (0.00–5.00)	0.27 ± 0.96 (0.00–4.00)	–	–	0.84
*UPDRS IV Dyskinesia* [No/Yes]	–	31 (96.9)/1 (3.1)	24 (92.3)/2 (7.7)	–	–	0.53
*UPDRS IV Fluctuation* [No/Yes]	–	30 (93.8)/2 (6.3)	24 (92.3)/2 (7.7)	–	–	0.50
FOG-Q	–	0.69 ± 1.03 (0.00–4.00)	1.15 ± 1.43 (0.00–5.00)	–	–	0.16

Values are reported as mean ± standard deviation (range) or absolute and percentage frequency (%) for continuous and categorical variables, respectively. Differences between PD patients and healthy controls and between PD groups were assessed using one-way ANOVA (for continuous demographic and general clinical variables) and Chi-square test (for all categorical variables). *P*-values were adjusted for multiple comparisons.

*FOG-Q* freezing of gait questionnaire, *HC* healthy controls, *N* number, *PD* Parkinson’s disease, *PIGD* Postural Instability/Gait Disorder dominant phenotype, *TD* Tremor dominant phenotype, *UPDRS* Unified Parkinson’s Disease Rating Scale.

Bold values indicates statistical significant values.

### Mapping motor activation in the cerebellum

In 23 healthy participants, fMRI activation of cerebellum during motor task (alternate dorsal/plantar foot flexion movements) is represented in Supplementary Fig. 1. The activation pattern was largely symmetrical in both cerebellar hemispheres with clusters of activity including lobules I–IV, V, VI and VIIIA/B bilaterally and vermis VI.

### Functional connectivity reorganization in PD subtypes

#### Healthy controls vs PD-TD

**Fig. 2:**
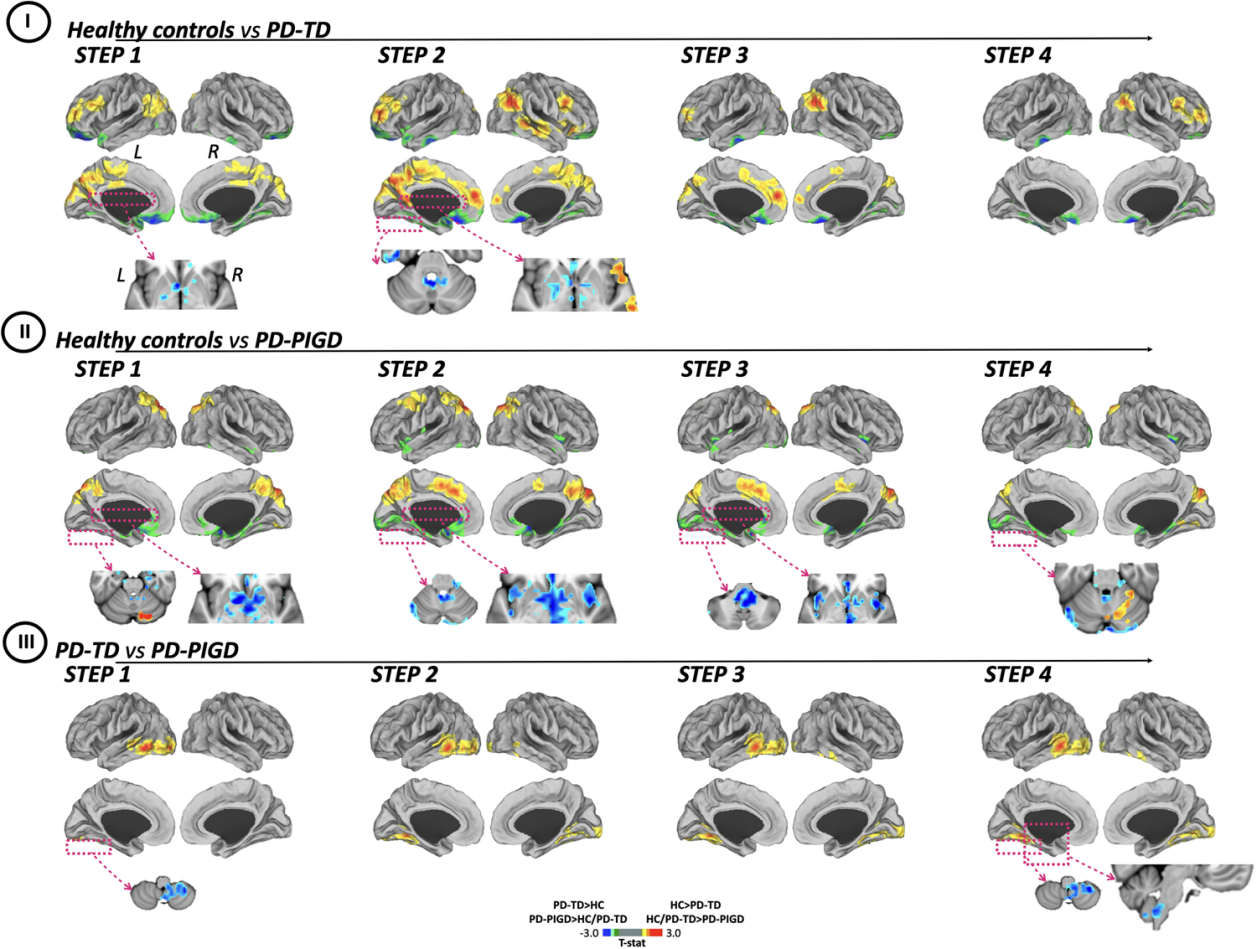
Differences between Parkinson’s disease participants and healthy controls in stepwise functional connectivity of the cerebellar seed region. Cortical maps represent the significant differences in stepwise functional connectivity values between PD subtypes and healthy controls (**I**, **II**) and among PD groups (**III**). Statistical analysis was adjusted for age and gender. Results were corrected for multiple comparisons using a threshold-free cluster enhancement method combined with nonparametric permutation testing at *p*-value <0.05 FWE-corrected. Color bars show the t-statistic applicable to the image. HC, healthy controls; L, left; R, right; PD-TD, Parkinson’s disease tremor dominant; PD-PIGD, Parkinson’s disease with postural instability and gait disorders.

At one-link step distance from cerebellar seed, PD-TD patients showed decreased connectivity in frontal lobe (middle frontal gyrus and dorsal-lateral prefrontal cortex), sensorimotor cortex (paracentral lobule), parietal lobe (posterior cingulate cortex, left inferior parietal cortex and precuneus) and cuneus relative to controls (Fig. 2-I and Supplementary Table 5). Across 2–4 link-steps, reduced SFC was found in frontal lobe (superior frontal, rostral middle frontal gyri, and anterior cingulate cortex), sensorimotor cortex (paracentral lobule), parietal (precuneus, right supramarginal gyrus, right inferior parietal, and isthmus cingulate cortices) and temporal lobe (right superior and right middle temporal gyri) and cuneus (Fig. 2-I and Supplementary Table 5). With regard to the opposite contrast, PD-TD patients exhibited significantly increased SFC at one-link step distance relative to controls in medial orbitofrontal gyri, temporal lobe (left superior temporal and right inferior temporal gyri and parahippocampal and entorhinal cortex), lingual gyrus, and subcortical regions (left putamen and globus pallidus). Across 2–4 link-steps, enhanced SFC was found in PD-TD patients in medial orbitofrontal gyri, temporal lobe (left superior temporal and left inferior temporal gyri), lingual gyrus, basal ganglia (left putamen and caudate, right globus pallidus, and subthalamic nucleus), cerebellum (lobule IX), and ponto-medullary junction (Fig. 2-I and Supplementary Table 5) relative to controls.

#### Healthy controls vs PD-PIGD

At one-link step distance, PD-PIGD patients presented lower direct connectivity in superior parietal cortex, precuneus, and cerebellum (Crus I) compared to controls (Fig. 2-II and Supplementary Table 5). At indirect link-step distances, PD-PIGD patients showed a lower SFC in frontal lobe (superior frontal and left caudal middle frontal gyri and left caudal anterior cingulate cortex), parietal lobe (superior parietal cortex and precuneus), sensorimotor cortex (left precentral and right paracentral gyri) and cuneus (Fig. 2-II and Supplementary Table 5). Referring to the opposite contrast, PD-PIGD patients exhibited enhanced SFC at one-link step distance from cerebellar seed to inferior frontal and temporal regions (parahippocampal gyrus, entorhinal, and orbitofrontal cortices) and subcortical regions (globus pallidus, right thalamus and medulla, as well as right lobules I–IV and VIII) compared to controls (Fig. 2-II and Supplementary Table 5). Across 2–4 link-steps, PD-PIGD patients presented enhanced SFC in orbitofrontal cortex, parahippocampal gyrus, insula, occipital lobe (left lingual gyrus and pericalcarine cortex), basal ganglia (putamen and thalamus) and cerebellum (lobules I–IV and from VIII to IX bilaterally) compared to controls (Fig. 2-II and Supplementary Table 5).

#### PD-TD vs PD-PIGD

When comparing PD patient motor subtypes at one-link step distance from cerebellar seed, PD-PIGD patients showed decreased SFC in temporal lobe (left middle and inferior temporal gyri) and left lateral occipital cortex relative to PD-TD cases (Fig. 2-III and Supplementary Table 5). Across 2–4 link-steps, PD-PIGD patients were characterized by additional decreased SFC within left fusiform gyrus and occipital lobe (right lingual gyrus and pericalcarine cortex) relative to PD-TD group (Fig. 2-III and Supplementary Table 5). Furthermore, at one-link step distance PD-PIGD patients showed increased SFC relative to PD-TD cases within the cerebellar cortex (from right lobules VIII to IX). At intermediate link-step distances, PD-PIGD patients were characterized by additional increased SFC in ponto-medullary junction relative to PD-TD patients.

### Subtype-conversion analysis

#### Demographic and clinical results

Since PD-TD may convert into PD-PIGD along the disease progression, longitudinal clinical data of PD-TD cases were examined. PD-TD subjects were divided in those who converted to PIGD over 4-year follow-up (cPD-TD) and those who did not (ncPD-TD). Four-year clinical follow-up data were available for 28 PD-TD patients: 10 subjects convert to PIGD over 4-year follow-up, while 18 PD-TD remain stable over time (Table 2 and Supplementary Tables 2 and 3). Four PD-TD subjects presented inconsistences in the longitudinal clinical data reports over time and were excluded from the analysis. There were no significant demographic differences between cPD-TD and ncPD-TD subtypes in terms of age, sex, education, and handedness at baseline (Table 2). Clinical characteristics of the two groups differed only for Hoehn & Yahr, UPDRS total, Bradykinesia and Rigidity UPDRS-III subscores and Freezing of Gait Questionnaire (FOG-Q) score (Table 2). No cognitive differences were found between the two subgroups of patients (Supplementary Table 2).

**Table 2:** Demographic and clinical characteristics of healthy controls, cPD-TD and ncPD-TD patients.

Variables	HC	ncPD-TD subtype	cPD-TD subtype	p:HC *vs* ncPD-TD	p:HC *vs* cPD-TD	p:ncPD-TD *vs* cPD-TD
*N*	60	18	10			
*Demographic and general clinical variables*						
Age at MRI [years]	61.79 ± 8.9 (46.14–77.72)	60.31 ± 7.98 (43.43–75.87)	60.69 ± 8.22 (46.46–73.53)	1.00	1.00	1.00
Sex [men/women]	29 (48.3)/31 (51.7)	12 (70.0)/6 (30.0)	4 (70.0)/6 (30.0)	0.91	0.74	0.24
Education [years]	13.52 ± 2.57 (8.00–16.00)	13.17 ± 2.77 (7.00–20.00)	12.30 ± 2.75 (8.00–16.00)	1.00	0.56	1.00
Handedness [right/left/both]	46 (92.0)/4 (8.0)/0 (0.0)	18 (100.00)/0 (0.0)/0 (0.0)	9 (90.0)/1 (10.0)/0 (0.0)	0.57	1.00	0.36
Age at onset [years]	–	58.75 ± 7.74 (42.50–73.00)	57.90 ± 8.16 (42.00–70.00)	–	–	0.79
PD duration [years]	–	1.56 ± 1.49 (0.14–5.30)	2.79 ± 2.11 (0.28–7.58)	–	–	0.08
Family history [No/Yes]	–	13 (72.2)/5 (27.8)	7 (70.0)/3(30.0)	–	–	1.00
Side of onset [right/left/both]	–	12 (66.7)/6 (33.3)/0(0.0)	4 (40.0)/3 (30.0)/0 (0.0)	–	–	0.24
Levodopa equivalent daily dose [mg]	–	179.17 ± 208.10 (0.00–740.00)	383.00 ± 342.38 (0.00–970.00)	–	–	0.06
*Clinical motor variables*						
Hoehn & Yahr	–	1.03 ± 0.12 (1.00–1.50)	1.45 ± 0.76 (1.00–3.00)	–	–	**0.03**
UPDRS total	–	25.17 ± 9.28 (11.0–47.00)	38.00 ± 22.02 (12.00–81.00)	–	–	**0.04**
UPDRS II Total	–	5.11 ± 4.13 (0.00–19.00)	7.30 ± 5.01 (0.00–16.00)	–	–	0.22
UPDRS III Total	–	15.89 ± 3.36 (10.00–23.00)	23.70 ± 16.55 (10.00–52.00)	–	–	0.06
*UPDRS III Axial*	–	1.39 ± 0.92 (0.00–3.00)	2.20 ± 2.25 (0.00–7.00)	–	–	0.19
*UPDRS III Bradykinesia*	–	5.5 ± 1.65 (3.00–8.00)	11.20 ± 8.09 (5.00–25.00)	–	–	**0.01**
*UPDRS III Rigidity*	–	2.22 ± 0.73 (0.00–3.00)	3.60 ± 2.56 (1.00–8.00)	–	–	**0.04**
UPDRS IV Total	–	0.00 ± 0.00 (0.00–0.00)	0.70 ± 1.64 (0.00–5.00)	–	–	0.08
*UPDRS IV Dyskinesia* [No/Yes]	–	18 (100)/0 (0)	9 (90)/1 (10)	–	–	0.36
*UPDRS IV Fluctuation* [No/Yes]	–	18 (100)/0 (0)	8 (80)/2 (20)	–	–	0.24
FOG-Q	–	0.28 ± 0.46 (0.00–1.00)	1.50 ± 1.43 (0.00–4.00)	–	–	**0.002**

Values are reported as mean ± standard deviation (range) or absolute and percentage frequency (%) for continuous and categorical variables, respectively. Differences between PD patients and healthy controls and between PD-TD subtypes were assessed using one-way ANOVA (for continuous demographic and general clinical variables) and Chi-squared test (for all categorical variables). *P*-values were adjusted for multiple comparisons.

*c* converter, *FOG-Q* freezing of gait questionnaire, *HC* healthy controls, *N* number, *nc* non-converter, *PD* Parkinson’s disease, *TD* Tremor dominant phenotype, *UPDRS* Unified Parkinson’s Disease Rating Scale.

Bold values indicates statistical significant values.

#### Connectivity reorganization in PD-TD converters and non-converters to PIGD

##### ncPD-TD vs cPD-TD

**Fig. 3:**
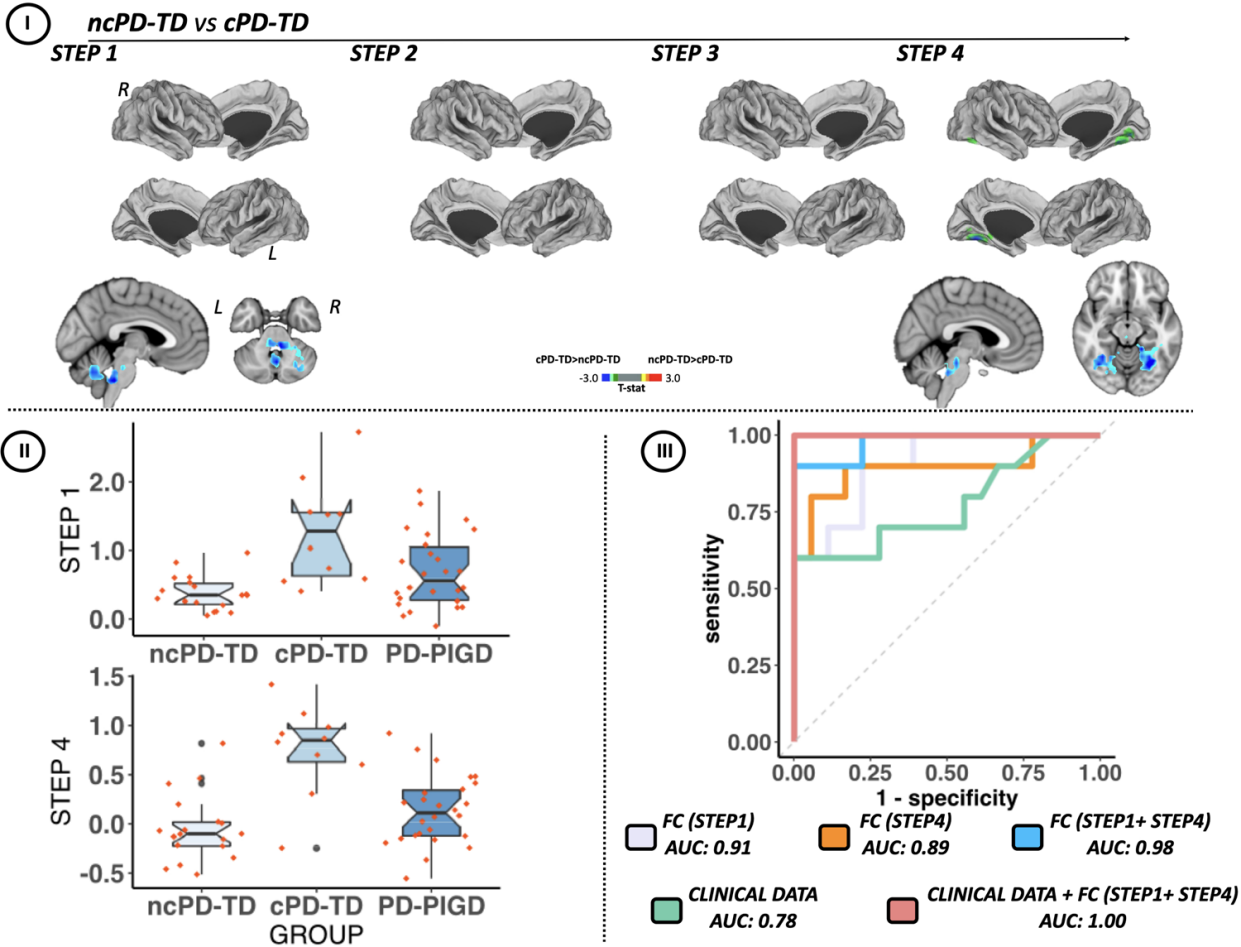
Differences in stepwise functional connectivity of the cerebellar seed region between PD-TD converters and non-converters to PD-PIGD subtype in a 4-year follow-up. **I** Cortical maps represent the significant differences in stepwise functional connectivity values between PD-TD subtypes. Statistical analysis was adjusted for age and gender. Results were corrected for multiple comparisons using a threshold-free cluster enhancement method combined with nonparametric permutation testing at *p*-value < 0.05 FWE-corrected. Color bars show the t-statistic applicable to the image. **II** Functional connectivity of altered network in PD-TD converters to PIGD subtype. Box plot of functional connectivity of altered brain network is shown for patient group. The red horizontal line in each box plot represents the median, the two lines just above and below the median represent the 25th and 75th percentiles, whiskers represent the minimum and maximum values, and all the dots outside the confidence interval are considered as outliers. **III** ROC analyses for characterization of patient at high risk of conversion to PIGD subtype. The predictive accuracy of direct (one-link-step) and indirect (four link-steps) functional connectivity and/or clinical data for the conversion to PIGD subtype was evaluated by area under the curve of ROC analysis. AUC, area under the curve; c, converters; CI, confidence interval; FC, functional connectivity; HC, healthy controls; L, left; nc, non-converter; R, right; ROC, receiver operating characteristic; PD-TD, Parkinson’s Disease tremor dominant.

When comparing PD-TD patient subtypes, at one-link step distance from cerebellar seed, cPD-TD patients showed increased SFC within cerebellar cortex (lobules left V–VI and Crus I, right I–IV and from V to VI) and in ponto-medullary junction relative to ncPD-TD cases (Fig. 3-I and II and Supplementary Table 6). At four link-step distance, cPD-TD patients were characterized by additional increased SFC in temporal lobe (fusiform and left parahippocampal gyri), occipital lobe (lingual gyri and lateral occipital cortex) and within cerebellar cortex and in ponto-medullary junction relative to ncPD-TD group (Fig. 3-I and -II and Supplementary Table 6).

##### c/ncPD-TD vs PD-PIGD

PD-PIGD patients presented lower direct SFC in left fusiform gyrus, superior, middle, and inferior temporal gyri only compared to cPD-TD (Supplementary Figure 2-I and Supplementary Table 6). Across 2–4 link-steps, PD-PIGD patients showed a lower SFC in temporal and occipital lobes relative to both cPD-TD and ncPD-TD groups (Supplementary Figure 2-I and II and Supplementary Table 6). In addition, PD-PIGD patients showed an increased SFC in cerebellar cortex only relative to ncPD-TD patients (Supplementary Figure 2-II and Supplementary Table 6).

#### ROC analyses for characterization of PD-TD patients at risk of conversion to PIGD

Clinical and SFC data that differed at study entry between cPD-TD and ncPD-TD entered the analysis. Clinical features were Hoehn & Yahr, UPDRS total and FoG-Q score. One (direct) and four (indirect) link-steps differences have been inserted in the analysis, first separately and then together. ROC curve showed that clinical data alone predict conversion to PIGD subtype with an area under the curve (AUC) of 0.78 (95%CI: 0.80–1) (Fig. 3-III). SFC data alone had a high power to discriminate cPD-TD from ncPD-TD: AUC for direct connectivity, 0.91 (95%CI: 0.80–1); AUC for indirect connectivity, 0.89 (95%CI: 0.74–1); AUC for combined direct and indirect, 0.98 [(95%CI: 0.80–1) (Fig. 3-III). Combining clinical and SFC data, ROC curves provided the highest classification power to discriminate cPD-TD from ncPD-TD (AUC, 1 [(95%CI: 1–1)]) (Fig. 3-III).

## Discussion

Obscured by basal ganglia and not part of Braak’s staging model, cerebellar involvement has only recently emerged as an integral component of PD pathophysiology, influencing the clinical presentation and natural history of the disease^20,21^. In this study, we identified patterns of functional network reorganization starting from cerebellar motor regions in PD-TD and PD-PIGD patients using novel graph theory metrics (i.e., SFC analysis). SFC revealed subtype-specific PD changes in cerebellar connectivity across both cortical and subcortical networks. Combining clinical and SFC data also provided a model to predict the conversion from PD-TD to PD-PIGD.

Considering that TD/PIGD classification is based on motor symptoms^2^, we aimed to identify functional motor network reorganization using SFC analysis starting from purely motor cerebellar seed regions. Instead of using known whole motor cerebellar lobules based on previous studies, we obtained motor cerebellar seeds associated with a task-based fMRI in a separate group of healthy controls^13,22^. Activation pattern included cerebellar lobules I–IV, V, VI and VIIIA/B bilaterally and vermis VI, all known to be recruited while engaging in purely motor tasks^23,24^. Starting from motor cerebellar areas we investigated functional motor reorganization between cerebellum and cortical/subcortical regions in different PD motor subtypes using SFC analysis. Conventional strategies as well as more-sophisticated graph theory analyses previously showed that PD motor subtypes are characterized by hypo- and hyper-connectivity patterns^11^. However, only few studies investigated the role of cerebellum in PD using graph analysis and connectomics^25^ and, to the best of our knowledge, only one in the two motor subtypes^19^. In the present study, the SFC approach was able to provide information regarding nodal properties of network geodesic distance of voxels with respect to the rest of the voxels in the entire network (and not just their strength^19^). With such a framework, our results confirm and expand the knowledge of motor cerebro-cerebellar network involvement in PD pathophysiology^11,13,15,22,26^. Notably, these results might lay the groundwork for future prognostic models that include neuroimaging biomarkers and provide possible targets for new therapeutics.

Functional network alterations within cerebro-cerebellar motor pathways were found between controls and PD-TD and PD-PIGD subtypes and among each other. In line with previous literature, compared to controls PD patients presented increased connectivity between cerebellum and basal ganglia^8^, in agreement with the strict interconnections between these structures^27^, and decreased SFC in several hub regions including the cuneus, parietal, sensorimotor, temporal, and frontal networks. These hubs are known to be susceptible to the accumulation of α-synuclein leading to functional disconnection between regions^28–30^ and to be associated with specific PD symptoms^31^. Only PD-PIGD group reported a SFC disruption in the precentral gyrus (which is involved in postural control, balance and gait^8,32^) and increased SFC in the insular cortex (known to display increased activation in gait-related task in PD patients^33^). Together these findings are consistent with gait impairment and other clinical manifestations of PD-PIGD patients.

Both PD motor subtypes exhibited an increased SFC within posterior occipital-temporal cortex relative to controls (Fig. 2-III). It has previously been proposed that posterior cortical network activity may act as an early compensatory mechanism in the generation of motor plans^34,35^. Comparing the two subtypes, PD-PIGD group presented a lower connectivity relative to PD-TD but still higher than controls. As already highlighted by previous results, PD-PIGD phenotype may represent a more aggressive disease and consequently show an initial functional deterioration of this compensatory mechanism.

Both PD motor phenotypes exhibited enhanced SFC within cerebellar motor lobules relative to controls, at direct and indirect link-step distances in line with current evidence on the role of the cerebellum in PD^15,36^. Moreover, PD-PIGD showed higher cerebellar connectivity compared to PD-TD, reflecting both an increased pathogenic and compensatory mechanism at least in the early stage of the disease. Compared to controls, PD-PIGD patients presented altered connectivity in the Cerebellar Crus I^37^ and pedunculopontine nucleus^38^, which are known to be implicated in the pathogenesis of FOG due to their “hybrid” cognitive and motor nature^39^. This is consistent with the higher FOG-Q scores of these patients in our sample (that presumably did not reach statistical significance due to the early stage of the disease in our cohort) and overall with the PD-PIGD features. Furthermore, reduced Crus I (cognitive-related subregion) activity in PD-PIGD compared to PD-TD patients was associated with cognitive dysfunction^8^.

Analyzing longitudinal data, a subgroup of PD-TD patients converting to PIGD subtype was identified. Compared to clinical features including disease severity, disability, and FOG-Q score, neuroimaging findings (increased SFC between cerebellar regions and occipital-temporal cortex) showed higher power to predict clinical conversion (Fig. 3-III). In the early stage of the disease, compensatory mechanism might be enhanced to counteract the more severe pathogenic mechanism, explaining why those patients still appeared as TD at baseline despite of the “smoldering” PIGD conversion. In our hypothesis, when the compensatory mechanism is no longer effective, patients convert to the PIGD phenotype and connectivity progressively decreases. Longitudinal neuroimaging follow-up is needed to confirm this model. This finding is particularly relevant to clinical practice, as the prediction of transition to PIGD carries important information. Impairment of gait and balance is a major determinant of autonomy, quality of life, and risk of falls in PD, as well as the care-givers’ burden and social costs of the disease^3^. Early recognition of patients at risk of converting to PIGD might lead to early implementation of fall prevention strategies that are known to be more effective in earlier stages of the disease^22^. In addition, PD-PIGD motor subtype is associated with an increased risk of dementia^5,40,41^, additional non-motor symptoms^41^, and higher rates of mortality^40^. Although it is not currently possible to address high-risk patients to preventive strategies, it appears clear that the transition to this phenotype identifies those patients who require closer surveillance and provides important prognostic information.

The study is not without limitations. First, the classification of PD motor subtypes in TD/indeterminate/PIGD put forward by Jankovic is inherently unstable^2^, as one subject can shift forward and back to one subtype to another along disease progression^4,12^. In addition, it is possible that the two motor subtypes do not represent fixed entities, but rather represent different stages of disease progression. To partially address this issue, in our study we included patients in the early phase of the disease and used a matching analysis, in which each PD-TD subject was matched to a PD-PIGD case with the same values on all covariates (age, sex, education, UPDRS-III, and disease duration). Subsequently, fMRI was only analyzed at baseline. Additional longitudinal connectivity studies are needed to assess whether functional brain organization changes differently with disease progression related to these two PD motor subtypes. Finally, the study of cerebellar structure and function in neuroimaging is limited, due to the innate low-resolution of the standard structural MRI, specifically in 1.5 T.

In conclusion, our data suggest functional network changes according to PD motor subtypes starting from motor cerebellar networks. Our results identified subtype-specific PD changes in cerebellar SFC across both cortical and subcortical networks. Taken together, our results pave the way toward better understanding of the cerebellum role in PD pathophysiology and different motor PD motor subtypes offering important insights in understanding the mechanism of disease and developing new therapeutic approaches.

## Methods

### Participants

One hundred-fifty-four patients with idiopathic PD were prospectively recruited at the Clinic of Neurology, Faculty of Medicine, University of Belgrade, Serbia, within the framework of an ongoing longitudinal project. Patients were included regardless of age, sex, education, family history of PD, age at disease onset, disease duration, side of onset, and levodopa equivalent daily dose (LEDD)^42^. Patients were excluded if they had: Hoehn and Yahr^43^ stage score >4; moderate/severe head tremor at rest; known PD-related genetic mutations (i.e., parkin and leucine-rich repeat kinase 2); dementia^44^; cerebrovascular disorders or intracranial masses on routine MRI; history of traumatic brain injury; any other major neurological and medical condition. Patients were assessed by clinical, cognitive/behavioral, and brain MRI evaluations (T1-weighted and resting-state functional MRI [rs-fMRI]) at baseline and clinically every year for a maximum of 4 years^45,46^. Eight PD patients were excluded from analysis due to incomplete MRI or movement artifacts.

An experienced neurologist blinded to MRI results performed clinical assessments^45,46^. Patients were examined in ON state (i.e., period when dopaminergic medication is working and symptoms are well controlled). Demographic, general clinical, and family data (sex, education, age, handedness, age at onset, side of onset, PD duration, and family history) were obtained using a semi-structured interview. LEDD was calculated. Disease severity was defined using the HY stage score and UPDRS. UPDRS was used to evaluate non-motor symptoms (UPDRS I), motor symptoms (UPDRS II), motor signs (UPDRS III) and motor complications (UPDRS IV). UPDRS III rigidity, axial, and bradykinesia subscores were also calculated. Severity of (FOG) was assessed using the FOG questionnaire. The presence of hallucinations was reported according to the UPDRS I subscore and of dyskinesia and fluctuations according to the UPDRS IV subscores. Cognitive and behavioral assessment was performed as previously described^45,46^. Non-motor symptoms other than cognitive impairment (gastrointestinal, urinary, olfactory, orthostatic and sexual dysfunctions) were assessed according to the Non-Motor Symptoms questionnaire (NMS-Q).

Sixty age- and sex-matched healthy controls without any neurological, psychiatric, or other disorders were also recruited among friends and relatives of patients and by word of mouth and performed the same study protocol.

Ethical standard committees on human experimentation of IRCCS Ospedale San Raffaele, Milan, Italy and Clinic of Neurology, Faculty of Medicine, University of Belgrade, Serbia approved the study protocol; all participants provided written informed consent prior to study inclusion.

### PD motor subtypes definition

PD patients were categorized in two motor subtypes (PD-TD and PD-PIGD) using the Movement Disorder Society Unified Parkinson’s Disease Rating Scale (MDS-UPDRS)^47^ as previously described^2^. Starting from the original sample (146 PD patients), 49 cases were classified as PD-TD and 83 as PD-PIGD, while 14 indeterminate patients were discarded from the analysis. We made the two PD groups more comparable to capture biologically relevant neuroimaging differences and avoid possible biases such as a disproportionate disease duration in PD-PIGD subjects. An “exact” matching analysis was applied in which age, sex, education, disease severity (UPDRS-III) and disease duration were used as covariates in PD group matching. Two groups of 32 PD-TD and 26 PD-PIGD patients were identified (Table 1 and Supplementary Tables 1 and 3). Analysis was performed using MatchIt package in R Statistical Software (version 4.0.3; R Foundation for Statistical Computing, Vienna, Austria).

Since PD-TD may convert into PD-PIGD along the disease progression, longitudinal clinical data of PD-TD cases were examined. PD-TD subjects were divided in those who converted to PIGD over 4-year follow-up (cPD-TD) and those who did not (ncPD-TD). PD-TD subjects presenting inconsistences or missing data in the clinical data reports during follow-up were excluded from this subanalysis.

### MRI analysis

#### Mapping motor activation in the cerebellum

Twenty-three right-handed independent healthy controls were recruited at the San Raffaele Scientific Institute by word of mouth and performed neuropsychological and MRI assessments (Supplementary Table 4). Subjects were excluded if they had: medical illnesses or substance abuse that could interfere with cognition; any major systemic, psychiatric, neurological, visual, and musculoskeletal disturbances; contraindications to undergo MRI examination; brain damage at routine MRI, including lacunae and extensive cerebrovascular disorders.

Healthy participants underwent a neuropsychological assessment. A blinded and experienced neuropsychologist performed a comprehensive cognitive evaluation including: Mini-Mental State Examination (MMSE)^48^; Digit span forward^49^ and Rey Auditory Verbal Learning Test (RAVLT) immediate and delayed recall^50^ and recognition^51^; Modified Card Sorting Test^52^; phonemic and semantic verbal fluency tests^53^; Attentive Matrices Test^54^; Trail Making Test (TMT)^55^ and digit span backward^56^; Freehand copying of drawings with and without landmarks^50^; Token Test^57^; Beck depression inventory (BDI)^58^; Apathy Rating Scale^59^; Snaith-Hamilton Pleasure Scale (SHAPS)^60^.

Brain MR images were acquired using a 3.0 Tesla Philips Intera scanner (Ingenia CX, Philips Medical Systems, Best, The Netherlands). The following sequences were acquired in all subjects: (i) three-dimensional (3D) T1-weighted sequence (repetition time (TR) = 7.1 ms, echo time (TE) = 3.2 ms, flip angle = 9°, 204 contiguous sagittal sections, thickness = 1 mm, field of view (FOV) = 256 × 240 mm, matrix = 256 × 240, voxel reconstruction 1 1 × 1 mm); (ii) 3DT2-weighted sequence (TR = 2500 ms, TE = 330 ms, flip angle = 90°, 192 contiguous sagittal sections, thickness = 1 mm, FOV = 256 × 256 mm, matrix = 256 × 258, voxel reconstruction 0.9 × 0.9 × 1 mm); and (iii) fMRI using a T2* weighted echo planar imaging (EPI) sequence (TE = 35 ms, TR = 1572 ms, flip angle = 70°, FOV = 240 × 240 mm, matrix = 96 × 94, 48 contiguous axial sections, thickness = 3 mm, acquisition time = 3 min and 57 s, voxel reconstruction 2.5 × 2.5 × 3 mm). fMRI scans were acquired during the performance of a “motor-task”, which consisted in alternated self-paced dorsal and plantar flexion movements of the feet with eyes closed, with the knees supported by a soft wedge and flexed of about 30°. To standardize the amplitude of movements, subjects were asked to reach a fixed wood bar with their insteps so that the dorsal flexion was about 90°. A block design (ABAB) was used, in which the activation A (lasting about 19 s) corresponded to the dorsal and plantar flexion, while during the resting period B (lasting about 19 s) subjects were asked to maintain their eyes closed, and each period was repeated 6 times (total duration 3′57″). Subjects performed the task according to auditory stimuli (“go” and “stop”) at the beginning and at the end of the movement. A visual signal (green/red light), visible to the operators outside the MRI scanner, was projected in order to monitor the correct time of task execution. Subjects were trained to perform the task outside the scanner and were carefully monitored visually by an observer inside the scanner room during acquisition to ensure a correct performance. Tasks were performed well by all the subjects.

Cerebellums were isolated from 3DT1-weighted images using the SUIT toolbox in SPM12 software (http://www.fil.ion.ucl.ac.uk/spm, Wellcome Trust Center for Neuroimaging, London). Individual white and gray matter segmented maps of cerebellum were then normalized into the SUIT atlas template using Dartel space. The normalization process uses the tissue segmentation maps, the white and gray matter segmentation maps resulting from the previous step of isolation of the cerebellum. The flowfield and affine transformations resulting from the normalization process were then used to bring different images from the anatomical space into SUIT space.

Task-based fMRI data were analyzed using SPM12 software. For each participant, all the volumes of fMRI data were realigned to the first one to correct for head movement (all study participants showed maximal head movements lower than 3 mm in each direction). Realigned functional data were then coregistered to the anatomical T1 images. All first-level analyses were performed at single-subject level. Signal variations of the BOLD effect associated with the execution of motor-task were evaluated voxel by voxel running the first-level General Linear Model (GLM). Specific effects were tested applying appropriate linear contrasts. Significant hemodynamic changes for each contrast were evaluated using Statistical Parametric Maps “t” (SPMt). Contrast images, resulting from first-level analysis, were then resliced into SUIT space applying flowfield and affine transformations obtained from normalization step. fMRI data in the SUIT space were then smoothed applying a 4-mm 3D-Gaussian filter. Second-level analysis was performed on smoothed fMRI data. One-sample *t*-test in SPM12 was performed to evaluate significant mean brain activations of healthy controls. A segmented cerebellum mask of healthy subjects was used as inclusion mask. All findings are shown at *p* < 0.001 uncorrected at the voxel level but only clusters passing a small volume correction for multiple comparisons (10 mm radius), cut-off value for significance *p* < 0.05, were presented. Cerebellar activations, resulting from second-level analysis, resulted into a 2 × 2 × 2 mm^3^ space. Finally, activations were normalized to 5 × 5 × 5 mm^3^ space, in order to obtain the motor cerebellar seed regions for the SFC analysis (Fig. 1A).

#### Reconstruction of cerebro-cerebellar pathways using stepwise connectivity analysis

##### MRI acquisition

Brain MRI scans were acquired on the same 1.5 Tesla Philips Medical System Achieva machine. Subjects were scanned between 10 and 11 a.m., i.e., treated PD patients were 90–120 min after their regular morning dopaminergic therapy administration (ON state). The following MR sequences were obtained: (i) dual-echo (DE) turbo spin-echo (SE) (TR = 3125 ms, TEs = 20/100 ms, echo train length [ETL] = 6, 44 axial slices, thickness = 3.0 mm, matrix size = 256 × 247, FOV = 240 × 232 mm^2^; voxel size, 0.94 × 0.94 × 3 mm, in-plane sensitivity encoding [SENSE] parallel reduction factor, 1.5); (ii) 3D sagittal T1-weighted Turbo Field Echo (TFE) (frequency direction = anterior-posterior, TR = 7.1 ms, TE = 3.3 ms, inversion time = 1000 ms, flip angle = 8°, matrix size = 256 × 256 × 180 [inferior-superior, anterior-posterior], FOV = 256 × 256 mm^2^, section thickness = 1 mm; voxel size = 1 × 1 × 1 mm, out-of-plane SENSE parallel reduction factor = 1.5, sagittal orientation); and (iii) gradient-echo (GRE) EPI for rs-fMRI (TR = 3000 ms, TE = 35 ms, flip angle = 90°, matrix size = 128 × 128, FOV = 240 × 240 mm^2^; slice thickness = 4 mm, 200 sets of 30 contiguous axial slices). During RS fMRI scanning, subjects were instructed to remain motionless, to keep their eyes closed, and not to think about anything in particular.

##### Image preprocessing

rs-fMRI data processing was performed with the Data Processing Assistant for Resting-State toolbox (DPARSFA, http://rfmri.org/DPARSF^61^), based on Statistical Parametric Mapping (SPM12, http://www.fil.ion.ucl.ac.uk/spm), and the rs-fMRI Data Analysis Toolkit [http://www.restfmri.net^62^]. Preprocessing included the following: (1) removal of first four volumes of each raw rs-fMRI dataset to allow for T1 equilibration; (2) slice timing correction for interleaved acquisitions (the middle slice was used as the reference point); (3) head motion correction using a six-parameter (rigid body) linear transformation with a two-pass procedure (registered to the first image and then registered to the mean of the images after the first realignment); (4) spatial normalization to the Montreal Neurological Institute (MNI) atlas template with voxel size was set at 5 × 5 × 5 mm^3^ for computational efficiency; (5) removal of spurious variance through linear regression: including 24 parameters from the head motion correction step [6 head motion parameters, 6 head motion parameters one time point before, and the 12 corresponding squared items^63^], scrubbing with regression [signal spike regression as well as 1 back and 2 forward neighbors^64^] at time points with a frame-wise displacement (FD) > 0.5 mm^65^, linear and quadratic trends, global signal, white matter signal, and the cerebrospinal fluid signal; (6) spatial smoothing with a 4 mm FWHM Gaussian Kernel; and (7) band-pass temporal filtering (0.01–0.08 Hz) to reduce the effect of low frequency drift and high frequency noise^66,67^.

No participant had more than 2 mm/degree of movement in any of the six directions, and no more than 90 volumes removed during scrubbing (1/3 of the total volumes), ensuring at least 5 min and 30 s of functional data per individual.

##### Stepwise functional connectivity analysis

SFC analysis is a graph theory metric that detects both direct and indirect functional couplings between a predefined seed region and other brain regions (Fig. 1B)^16,68,69^. SFC analysis aims to characterize regions connected to specific seed brain areas at different levels of link-step distances^16,68,69^. With such a framework, a step refers to the number of links (edges) that belong to a path connecting a node to the seed (or target) area. In SFC analysis, the degree of stepwise connectivity of a voxel *j* for a given step distance *l* and a seed area *i* ($A_{ji}^l$) is computed from the count of all paths that (1) connect voxel *j* and any voxel in seed area *i*, and (2) have an exact length of *l*. Each SFC matrix *A*_*l*_ of size m-by-m can be recursively represented as follows:

1 $$A_l\left( {i,j} \right) = \left\{ {\begin{array}{*{20}{c}} {A\left( {i,j} \right)\left[ {i \ne j,l = 1} \right]} \\ {\mathop {\sum}\limits_{k = 1}^m {\left( {\frac{{A_{l - 1}\left( {i,k} \right) - \min \left( {A_{l - 1}} \right)}}{{\max \left( {A_{l - 1}} \right) - \min \left( {A_{l - 1}} \right)}}} \right)\left( {\frac{{A\left( {k,j} \right) - \min \left( A \right)}}{{\max \left( A \right) - \min \left( A \right)}}} \right)\left[ {i \,\ne\, j,l \ge 2} \right]} } \end{array}} \right.$$

Here, *A*_*l*_ is the functional connectivity matrix with a step distance of *l*, and A is the correlation matrix after Fisher transformation. Matrices were then normalized between 0 and 1, keeping the final distribution of values intact while making them comparable across step distances. In this sense, a larger SFC degree under the step distance *l* indicates stronger paths connecting two voxels via link one, while a smaller degree indicates weaker connectivity paths. It is easy to see that SFC is also related to transition probabilities in an information flow analysis between the seed area and voxels across the rest of the brain. Given the lack of directionality information provided by rs-fMRI data, in SFC we did not include any restrictions about recurrent pathways crossing the seed regions multiple times. Therefore, we explored a wide range of link-step distances, from 1 to 20, to characterize the progression of the derived maps. However, as we found that the SFC patterns are topographically dissimilar between consecutive maps from steps one to three, and become stable for link-step distances above four, we only included maps from one to four steps in our results. SFC was applied to characterize pattern of rs-fMRI connectivity between the motor cerebellar seed region and the rest of the brain in PD-TD, PD-PIGD and matched 60 healthy controls (Fig. 1B).

##### Network construction

Association matrices for each participant were computed by calculating the Pearson correlation between each voxel time course and every other voxel time course within a mask covering cortical and subcortical gray matter. To perform this analysis, preprocessed resting state images of each participant were previously converted to an N-by-M matrix, where N was the image voxels in MNI space, and M was the 200 acquisition time points. From this step, a 11,705 × 11,705 matrix of Pearson correlation coefficients (*r*-values) was obtained for each individual. Fisher z transformation was applied to *r*-values. Then, all negative correlations and positive correlations that did not reach a false discovery rate (FDR) correction^67^ of *p*-value < 0.05 were excluded from further analyses. Final association matrix included only significant positive associations, as positive connectivity has been proved to drive functional connectivity network topology in the human brain.

### Statistical analyses

Demographic, clinical, and cognitive/behavioral data were compared between groups using ANOVA models (for continuous variables) or Chi-square test (for all categorical variables). Two-sided *p*-value < 0.05 was considered for statistical significance. *P*-values were adjusted for multiple comparisons (R Statistical Software).

In order to identify regions demonstrating between-group SFC differences, voxel-wise analyses were performed using general linear models as implemented in SPM12 (Wellcome Department of Imaging Neuroscience, London, England; www.fil.ion.ucl.ac.uk/spm). Whole-brain two-sample *t*-test comparisons between groups were performed, including age and sex as covariates. A threshold-free cluster enhancement method combined with nonparametric permutation testing (5000 permutations) as implemented in the Computational Anatomy Toolbox 12 (CAT12, http://www.neuro.uni-jena.de/cat/) was used to detect statistically significant differences at *p*-value < 0.05, family-wise error (FWE) corrected.

The discriminatory power of clinical data and/or SFC at different link-step distances in distinguishing cPD-TD from ncPD-TD patients was assessed using ROC curve analyses (Fig. 1C). Statistical significance level was set at *p*-value < 0.05. The area under the curve (AUC), as derived measure of accuracy, was calculated (R Statistical Software).

## Supplementary information

Supplementary materials

## Data Availability

The dataset used and analyzed during the current study will be made available by the corresponding author upon request to qualified researchers (i.e., affiliated to a university or research institution/hospital).
