# Supplementary material for: Cerebro-cerebellar motor networks in clinical subtypes of Parkinson’s disease
Source: NPJ Parkinsons Dis. 2022 Sep 6;8:113. doi: 10.1038/s41531-022-00377-w (PMC9448730; doi:10.1038/s41531-022-00377-w)
Supplement: Supplementary file 1 — Supplementary materials [file 41531_2022_377_MOESM1_ESM.pdf]

SUPPLEMENTARY MATERIALS

Supplementary Table 1. Cognitive and behavioural characteristics in healthy controls, PD-TD and PD-PIGD patients.

**Variables**	**HC**	**PD-TD subtype**	**PD-PIGD subtype**	**p: HC *vs* PD-TD**	**p: HC *vs* PD-PIGD**	**p: PD-PIGD *vs* TD**
**N**	60	32	26			
Global cognition						
**ACE-R total**	96.28 ± 3.00 (87.00-100.00)	90.72 ± 6.73 (72.00-99.0)	91.88 ± 6.45 (70.00-100.00)	**<0.001**	**0.048**	1.00
Memory						
**RAVLT, immediate recall**	45.36 ± 10.24 (21.00-65.00)	39.72 ± 11.46 (21.00-65.00)	38.58 ± 12.84 (0.00-59.00)	1.00	0.69	1.00
**RAVLT (A6), delayed recall**	8.92 ± 2.74 (4.00-13.00)	7.09 ± 3.05 (3.00-14.00)	7.40 ± 2.52 (3.00-12.00)	0.22	1.00	1.00
**PRM [% correct]**	79.69 ± 11.46 (37.50-95.83)	74.72 ± 14.22 (45.83-92.00)	77.92 ± 10.34 (50.00-100.00)	1.00	1.00	1.00
**SRM [% correct]**	73.88 ± 10.52 (55.00-95.00)	71.79 ± 13.35 (30.00-85.00)	70.22 ± 11.02 (35.00-85.00)	1.00	1.00	1.00
Language						
**BNT total**	58.22 ± 1.63 (53.00-60.00)	56.84 ± 3.60 (48.00-60.00)	56.64 ± 3.51 (44.00-60.00)	1.00	0.07	1.00
**ACE-R, language**	25.90 ± 0.30 (25.00-26.00)	24.88 ± 1.62 (21.00-26.00)	25.04 ± 2.03 (17.00-27.00)	**0.048**	0.46	1.00
Fluency						
**Semantic fluency**	19.60 ± 4.71 (12.00-30.00)	18.41 ± 6.05 (7.00-33.00)	19.04 ± 5.29 (11.00-29.00)	1.00	1.00	1.00
**Phonemic fluency**	37.94 ± 8.19 (20.00-59.00)	37.19 ± 9.30 (20.00-57.00)	36.84 ± 14.20 (13.00-63.00)	1.00	1.00	1.00
Executive functions						

**Digit backward**	6.80 ± 1.95 (3.00-11.00)	5.61 ± 1.80 (2.00-9.00)	5.88 ± 1.83 (3.00-11.00)	0.32	1.00	1.00
**Stroop, interference [total correct]**	38.24 ± 10.34 (17.00-60.00)	41.65 ± 16.87 (17.00-98.00)	38.32 ± 12.30 (13.00-70.00)	1.00	1.00	1.00
**IED [total errors]**	41.64 ± 28.99 (7.00-146.00)	48.32 ± 28.58 (15.00-162.00)	52.83 ± 28.35 (15.00-161.00)	1.00	1.00	1.00
Attention						
**Digit ordering [max. span]**	5.77 ± 1.24 (4.00-8.00)	5.83 ± 1.20 (4.00-8.00)	5.36 ± 1.00 (4.00-8.00)	1.00	1.00	1.00
**Letter cancellation correct**	28.44 ± 5.53 (20.00-41.00)	29.31 ± 6.90 (15.00-40.00)	28.83 ± 5.87 (20.00-39.00)	1.00	1.00	1.00
Visuospatial abilities						
**Hooper**	23.12 ± 3.34 (13.00-30.00)	22.92 ± 3.83 (12.00-30.00)	20.46 ± 4.95 (10.00-27.00)	1.00	0.30	0.96
**ACE-R, visuospatial**	15.80 ± 0.40 (15.00-16.00)	15.53 ± 1.08 (11.00-16.00)	15.52 ± 0.71 (14.00-16.00)	1.00	1.00	1.00
Mood/behaviour						
**Impulsive compulsive behaviour [No/yes]**	-	28 (87.5)/ 4 (12.5)	21 (80.8)/ 5 (19.2)	-	-	0.72
**HDRS**	2.30 ± 3.63 (0.00-15.00)	5.56 ± 5.88 (0.00-22.00)	6.69 ± 4.38 (0.00-15.00)	**0.02**	**<0.001**	1.00
**HAMA**	2.92 ± 3.06 (0.00-11.00)	4.75 ± 5.48 (0.00-21.00)	5.04 ± 3.14 (0.00- 10.00)	0.39	0.09	1.00
**Apathy scale**	1.74 ± 2.90 (0.00-11.00)	10.78 ± 8.59 (0.00-28.00)	12.08 ± 7.59 (0.00-28.00)	**<0.001**	**<0.001**	1.00

Values are reported as mean ± standard deviation (range) or absolute and percentage frequency (%) for continuous and categorical variables, respectively. Differences between PD patients and healthy controls and between PD groups were assessed using one-way ANOVA (for continuous variables) and Chi squared test (for all categorical variables). P-values were adjusted for multiple comparisons. Abbreviations: ACE-R=Addenbrooke's Cognitive Examination-Revised; BNT=Boston Naming Test; HAMA=Hamilton Anxiety Rating Scale; HC=Healthy Controls; HDRS=Hamilton Depression Rating Scale; IED=Intra and Extra-Dimensional shifting; N=Number; PD= Parkinson's disease; PIGD=Postural Instability/Gait Disorder

dominant phenotype; PRM= Pattern Recognition Memory; RAVLT=Rey Auditory Verbal Learning Test; SRM= Spatial Recognition Memory; TD=Tremor dominant phenotype.

Supplementary Table 2. Cognitive and behavioural characteristics of healthy controls, cPD-TD and ncPD-TD patients.

**Variables**	**HC**	**ncPD-TD subtype**	**cPD-TD subtype**	**p: HC *vs* ncPD-TD**	**p: HC *vs* cPD-TD**	**p: ncPD-TD *vs* cPD-TD**
**N**	60	18	10			
Global cognition						
**ACE-R total**	96.28 ± 3.00 (87.00-100.00)	90.83 ± 7.10 (72.00-99.0)	89.90 ± 7.56 (72.00-98.00)	**<0.001**	**0.02**	1.00
Memory						
**RAVLT, immediate recall**	45.36 ± 10.24 (21.00-65.00)	42.00 ± 12.26 (21.00-65.00)	36.60 ± 10.81 (21.00-51.00)	1.00	1.00	1.00
**RAVLT (A6), delayed recall**	8.92 ± 2.74 (4.00-13.00)	7.83 ± 3.55 (3.00-14.00)	6.10 ± 2.23 (3.00-10.00)	1.00	0.30	1.00
**PRM [% correct]**	79.69 ± 11.46 (37.50-95.83)	74.40 ± 11.42 (45.83-91.67)	82.55 ± 12.26 (54.00-92.00)	1.00	1.00	1.00
**SRM [% correct]**	73.88 ± 10.52 (55.00-95.00)	73.93 ± 8.81 (60.00-85.00)	75.50 ± 10.66 (55.00-85.00)	1.00	1.00	1.00
Language						
**BNT total**	58.22 ± 1.63 (53.00-60.00)	57.72 ± 2.59 (51.00-60.00)	55.20 ± 4.85 (48.00-60.00)	1.00	**0.03**	0.54
**ACE-R, language**	25.90 ± 0.30 (25.00-26.00)	25.28 ± 1.49 (21.00-26.00)	24.30 ± 1.89 (21.00-26.00)	1.00	<0.001	0.72
Fluency						
**Semantic fluency**	19.60 ± 4.71 (12.00-30.00)	19.56 ± 6.75 (7.00-33.00)	16.60 ± 5.19 (8.00-23.00)	1.00	1.00	1.00
**Phonemic fluency**	37.94 ± 8.19 (20.00-59.00)	40.39 ± 9.43 (20.00-57.00)	32.40 ± 7.01 (21.00-38.00)	1.00	1.00	0.85
Executive functions						
**Digit backward**	6.80 ± 1.95 (3.00-11.00)	5.61 ± 1.46 (3.00-9.00)	5.50 ± 2.46 (2.00-9.00)	1.00	1.00	1.00

**Stroop, interference [total correct]**	38.24 ± 10.34 (17.00-60.00)	40.18 ± 13.56 (22.00-78.00)	37.50 ± 9.64 (17.00-49.00)	1.00	1.00	1.00
**IED [total errors]**	41.64 ± 28.99 (7.00-146.00)	48.14 ± 36.90 (15.00-162.00)	45.60 ± 19.95 (15.00-65.00)	1.00	1.00	1.00
Attention						
**Digit ordering [max. span]**	5.77 ± 1.24 (4.00-8.00)	6.33 ± 1.20 (4.00-8.00)	5.05 ± 0.80 (4.00-6.00)	1.00	1.00	0.37
**Letter cancellation correct**	28.44 ± 5.53 (20.00-41.00)	31.33 ± 6.08 (22.00-40.00)	24.60 ± 6.96 (15.00-39.00)	1.00	1.00	0.22
Visuospatial abilities						
**Hooper**	23.12 ± 3.34 (13.00-30.00)	23.92 ± 3.47 (17.00-30.00)	21.30 ± 4.47 (12.00-28.00)	1.00	1.00	1.00
**ACE-R, visuospatial**	15.80 ± 0.40 (15.00-16.00)	15.39 ± 1.33 (11.00-16.00)	15.60 ± 0.70 (14.00-16.00)	1.00	1.00	1.00
Mood/behaviour						
**Impulsive compulsive behaviour [No/yes]**	-	16 (88.9)/ 2 (11.1)	8 (80.0)/ 2 (20.0)	-	-	0.60
**HDRS**	2.30 ± 3.63 (0.00-15.00)	3.83 ± 4.72 (0.00-17.00)	8.70 ± 7.63 (0.00-22.00)	1.00	**<0.001**	0.08
**HAMA**	2.92 ± 3.06 (0.00-11.00)	3.06 ± 3.21 (0.00-11.00)	7.80 ± 8.01 (0.00- 21.00)	1.00	**0.01**	**0.03**
**Apathy scale**	1.74 ± 2.90 (0.00-11.00)	10.28 ± 8.70 (0.00-28.00)	14.00 ± 8.84 (0.00-26.00)	**<0.001**	**<0.001**	0.90

Values are reported as mean ± standard deviation (range) or absolute and percentage frequency (%) for continuous and categorical variables, respectively. Differences between PD patients and healthy controls and between PD groups were assessed using one-way ANOVA (for continuous variables) and Chi squared test (for all categorical variables). P-values were adjusted for multiple comparisons. Abbreviations: ACE-R=Addenbrooke's Cognitive Examination-Revised; BNT=Boston Naming Test; c= converter; HAMA=Hamilton Anxiety Rating Scale; HC=Healthy Controls; HDRS=Hamilton Depression Rating Scale; IED=Intra and Extra-Dimensional shifting; N=Number; nc= non converter; PD= Parkinson's disease; PRM= Pattern Recognition Memory; RAVLT=Rey Auditory Verbal Learning Test; SRM= Spatial Recognition Memory; TD=Tremor dominant phenotype.

Supplementary Table 3. Non-motor symptoms of PD subtypes.

Variables	PD-TD subtype	PD-PIGD subtype	p: PD-PIGD vs PD-TD	ncPD-TD subtype	cPD-TD subtype	p: ncPD-TD *vs* cPD-TD
**N**	32	26		18	10	
**UPDRS I Total**	4.72 ± 4.36 (0.00-16.00)	5.65 ± 2.80 (0.00-11.00)	0.35	4.17 ± 4.20 (0.00-13.00)	6.30 ± 5.19 (0.00-16.00)	0.25
UPDRS I hallucinations and psychosis **[No/Yes]**	30 (93.8)/ 2 (6.3)	23 (88.5)/ 3 (11.5)	0.65	18 (100)/ 0 (0)	9 (90.0)/ 1 (10.0)	0.36
NMS-Q Gastrointestinal symptoms **[No/Yes]**	16 (50.0)/ 16 (50.0)	5 (19.2)/ 21 (80.8)	**0.03**	11 (61.1)/ 7 (38.9)	3 (30.0)/ 7 (70.0)	0.07
NMS-Q Urinary symptoms **[No/Yes]**	15 (46.9)/ 17 (53.1)	9 (34.6)/ 17 (65.4)	0.43	10 (55.6)/ 8 (44.4)	4 (40.0)/ 6 (60.0)	0.14
NMS-Q Olfactory dysfunction **[No/Yes]**	23 (71.9)/ 9 (28.1)	17 (65.4)/ 9 (34.6)	0.78	13 (72.2)/ 5 (27.8)	6 (60.0)/ 4 (40.0)	0.68
NMS-Q Sexual dysfunction **[No/Yes]**	19 (59.4)/ 13 (40.6)	13 (50.0)/ 13 (50.0)	0.60	10 (55.6)/ 8 (44.4)	6 (60.0)/ 4 (40.0)	0.68
NMS-Q Orthostatic symptoms **[No/Yes]**	27 (84.4)/ 5 (15.6)	17 (68.0)/ 8 (32.0)	0.21	16 (88.9)/ 2 (11.1)	7 (70.0)/ 3 (30.0)	0.32

Values are reported as mean ± standard deviation (range) or absolute and percentage frequency (%) for continuous and categorical variables, respectively. Differences between PD groups were assessed using one-way ANOVA (for continuous variables) and Chi squared test (for all categorical

variables). P-values were adjusted for multiple comparisons. Abbreviations: c= converter; N=Number; nc= non converter; NMS-Q= Non-Motor Symptoms Questionnaire; PD= Parkinson's disease; PIGD=Postural Instability/Gait Disorder dominant phenotype; TD=Tremor dominant phenotype; UPDRS=Unified Parkinson's Disease Rating Scale.

Supplementary Table 4. Demographic and cognitive/behavioural characteristics of healthy controls recruited for the definition of cerebellar motor seed region.

Variables	HC
**N**	23
**Age at MRI [years]**	63.75 ± 8.69 (52.05-80.08)
**Sex [Men/Women]**	10 (43.48)/ 13 (56.52)
**Education [Years]**	12.13 ± 3.72 (5.00-18.00)
**MMSE**	29.23 ± 0.87 (27.00-30.00)
Verbal Memory	
**RAVLT - Immediate recall**	49.50 ± 10.76 (29.00-67.00)
**RAVLT - Delayed recall**	10.41 ± 2.48 (6.00-14.00)
**RAVLT - Recognition**	14.18 ± 0.91 (12.00-15.00)
**Digit span forward**	6.32 ± 1.13 (4.00-8.00)
Language	
**Token Test**	33.73 ± 2.13 (28.00-36.00)
Visuospatial abilities	
**Copy of drawings - Freehand**	10.41 ± 1.05 (9.00-12.00)
**Copy of drawings with landmarks**	68.23 ± 2.13 (63.00-70.00)
Executive Functions, Attention And Working Memory	
**Attentive matrices**	54.0 ± 4.7 (40.00-60.00)
**Digit span backwards**	4.86 ± 1.17 (3.00-7.00)
**Trail making test A**	29.89 ± 9.72 (16.06-52.21)
**Trail making test B**	92.86 ± 33.16 (47.47-172.00)
**Trail making test B-A**	62.98 ± 27.36 (24.33-139.48)
**Phonemic fluency**	39.41 ± 8.16 (18.00-54.00)
**Semantic fluency**	50.41 ± 9.30 (26.00-65.00)
**MCST Categories**	4.40 ± 1.10 (3.00-5.00)
**MCST Perseverations**	3.68 ± 3.2 (1.00-5.50)

Mood	
**BDI**	7.50 ± 5.46 (0.00-20.00)
**Apathy rating scale**	8.00 ± 5.63 (0.00-19.00)
**SHAPS anhedonia**	0.30 ± 0.57 (0.00-2.00)
**SHAPS 16 items**	55.95 ± 3.95 (46.00-62.00)
**SHAPS 14 items**	49.30 ± 3.39 (40.00-54.00)

Values are reported as mean ± standard deviation (range) or absolute and percentage frequency (%) for continuous and categorical variables, respectively. Abbreviations: BDI= Beck Depression Inventory; HC= healthy controls; MCST= Modified Card Sorting test; MMSE= Mini Mental State Examination; N= number; RAVLT= Rey Auditory Verbal Learning Test; SHAPS= Snaith-Hamilton Pleasure Scale.

Supplementary Table 5. Cortical and subcortical regions showing functional connectivity differences across four link-step distances in PD-TD and PD-PIGD patients relative to healthy controls and between patient groups.

	STEP 1	***STEP 2***	***STEP 3***	***STEP 4***
<u>Healthy Controls vs PD-TD</u>				
↑ (yellow-red)	**L&R** middle frontal gyrus, precuneus, posterior cingulate cortex, paracentral gyrus and cuneus; **L** pars-orbitalis and pars-triangularis and inferior parietal cortex	**L&R** precuneus, cuneus, paracentral, superior frontal gyri; **L** isthmus cingulate cortex and rostral middle frontal gyrus**;** **R** inferior parietal cortex**,** superior and middle temporal gyri and pars-orbitalis	**L&R** anterior cingulate cortex and superior frontal gyrus; **L** middle frontal gyrus, cuneus and precuneus, **R** inferior parietal lobule, supramarginal gyrus and paracentral lobule	**R** rostral middle frontal and superior frontal gyri, inferior parietal cortex
↓ (green-blue)	**L&R** medial orbitofrontal cortex, entorhinal cortex and lingual gyrus; **L** superior temporal gyrus, **R** inferior temporal and parahippocampal gyri	**L&R** medial orbitofrontal cortex and lingual gyrus**;** **L** superior and inferior temporal gyri	**L&R** medial and lateral orbitofrontal cortices and fusiform gyrus; **L** superior and inferior temporal gyrus	**L&R** medial orbitofrontal cortex, fusiform and lingual gyri; **L** inferior temporal gyrus
Subcortical differences	↓ **L** Putamen and globus pallidus	↓ **L&R** Lobule IX**;** **L** putamen and caudate; **R** globus pallidus and subthalamic nucleus; ponto-medullary junction	-	-
<u>Healthy Controls vs PD-PIGD</u>				

↑ (yellow-red)	**L&R** precuneus and superior parietal cortex	**L&R** superior frontal gyrus, precuneus, superior parietal cortex and cuneus; **L** caudal anterior cingulate cortex, caudal middle frontal and precentral gyri	**L&R** precuneus and superior parietal cortex; **L** caudal anterior cingulate cortex and superior frontal gyrus; **R** paracentral gyrus	**L&R** precuneus and superior parietal cortex
↓ (green-blue)	**L&R** orbitofrontal and entorhinal cortices; **R** parahippocampal gyrus	**L&R** medial orbitofrontal cortex and parahippocampal gyri and insular cortex; **L** lingual gyrus and pericalcarine cortex	**L&R** medial orbitofrontal cortex, lingual and parahippocampal gyri and insular cortex; **L** pericalcarine cortex	**L&R** medial orbitofrontal cortex and parahippocampal gyrus; **L** lingual gyrus and pericalcarine cortex; **R** insular cortex
Subcortical differences	↓ **L&R** Globus pallidus; **R** Thalamus; lobules I-IV and VIII; ↑ **R** Crus I; Medulla	↓ **L&R** Thalamus, globus pallidus and putamen; Lobule VIII and IX; **L** Crus I; Medulla	↓ **L&R** Vermis and Lobule IX; **L** Crus I; **R** thalamus and globus pallidus; Medulla	↓ **L&R** Lobule I-IV and IX; **R** subthalamic nucleus; Medulla; ↑ **L&R** Crus I
PD-TD vs PD-PIGD				
↑ (yellow-red)	**L** middle and inferior temporal gyri, lateral occipital cortex	**L** fusiform gyrus, middle and inferior temporal gyri, lateral occipital cortex; **R** lingual gyrus and pericalcarine	**L** fusiform gyrus, middle and inferior temporal gyri and lateral occipital cortex; **R** lingual gyrus and pericalcarine	**L** fusiform gyrus, middle and inferior temporal gyri and lateral occipital cortex; **R** lingual gyrus and pericalcarine
↓ (green-blue)	-	-	-	-
Subcortical differences	↓ **R** Lobules VIII and IX	-	-	↓ **R** Lobules VIII and IX; ponto-medullary junction

Abbreviations: HC= healthy controls; L= left; PD-PIGD= Parkinson's Disease with postural instability and gait disorders; PD-TD= Parkinson's Disease tremor dominant; R= right.

Supplementary Table 6. Cortical and subcortical regions showing functional connectivity differences across four link-step distances in cPD-TD and ncPD-TD patients relative to PD-PIGD patients and among them.

	STEP 1	***STEP 2***	***STEP 3***	***STEP 4***
ncPD-TD vs cPD-TD				
↑ (yellow-red)	-	-	-	-
↓ (green-blue)	-	-	-	**L&R** fusiform and lingual gyri;<br>**L** parahippocampal gyrus;<br>**R** lateral occipital cortex
Subcortical differences	↓ **L** Lobules V-VI and Crus I;<br>**R** Lobules I-IV and from V to VI;<br>ponto-medullary junction	-	-	↓ **L** Lobules V-VI and Crus I;<br>**R** Lobules I-IV and from V to VI;<br>ponto-medullary junction
cPD-TD vs PD-PIGD				
↑ (yellow-red)	**L** fusiform gyrus, superior**,** middle and inferior temporal gyri	**L** fusiform gyrus, superior**,** middle and inferior temporal gyri, lingual gyrus and lateral occipital cortex	**L&R** fusiform gyrus, lateral occipital cortex and lingual gyrus;<br>**L** superior**,** middle and inferior temporal gyri	**L&R** fusiform gyrus, lateral occipital cortex and lingual gyrus;<br>**L** superior**,** middle and inferior temporal gyri;<br>**R** pericalcarine cortex

↓ (green-blue)	-	-	-	-
Subcortical differences	-	-	↑ **L** Lobules I-IV and from V to VI	↑ **L&R** Lobules I-IV and from V to VI and Crus II; vermis VI
<u>ncPD-TD vs PD-PIGD</u>				
↑ (yellow-red)	-	-	**L** fusiform gyrus, superior**,** middle and inferior temporal gyri and supramarginal gyrus	**L** fusiform gyrus, superior**,** middle and inferior temporal gyri and supramarginal gyrus
↓ (green-blue)	-	-	-	-
Subcortical differences	-	-	-	↓ **L** Lobules VIII a/b and X and Crus I and II; vermis VI

Abbreviations: c= converter; HC= healthy controls; L= left; nc= non converter; PD-PIGD= Parkinson's Disease with postural instability and gait disorders; PD-TD= Parkinson's Disease tremor dominant; R= right.

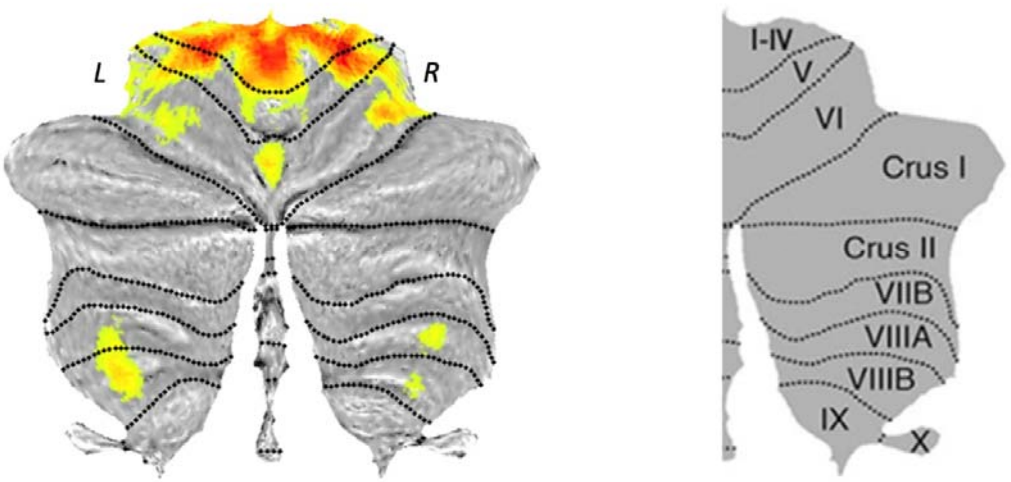

Supplementary Figure 1. Flat map of the fMRI cerebellar activations (SUIT, spatially unbiased atlas template of the cerebellum, https://www.diedrichsenlab.org/imaging/suit.htm). All activations are rendered with a threshold of $p < 0.001$ (corrected at cluster level). Legend indicating cerebellar lobules within a standard cerebellar flatmap is reported on the right side of the figure.

Abbreviations: L= left; R= right.

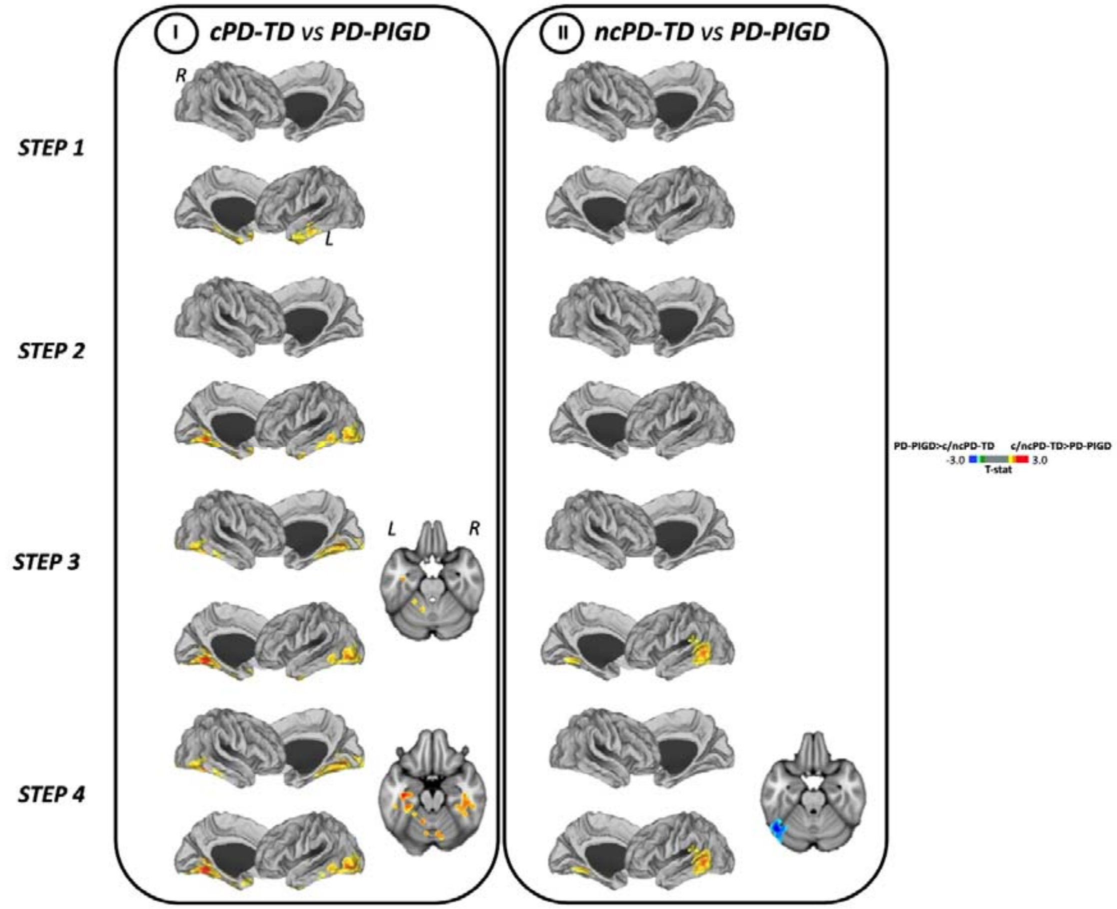

Supplementary Figure 2. Differences between converter and non converter PD-TD and PD-PIGD in stepwise functional connectivity of the cerebellar seed region. Cortical and subcortical maps represent the significant differences in stepwise functional connectivity values between PD-PIGD and cPD-TD subtypes (**I**) and ncPD-TD group (**II**). Statistical analysis was adjusted for age and gender. Results were corrected for multiple comparisons using a threshold-free cluster enhancement method combined with nonparametric permutation testing at $p<0.05$ FWE-corrected. Color bars show the t-statistic applicable to the image. Abbreviations: c= converter; HC= healthy controls; L= left; nc= non converter; R= right; PD-TD= Parkinson's Disease tremor dominant; PD-PIGD= Parkinson's Disease with postural instability and gait disorders.
